# Functionally Annotating Regulatory Elements in the Equine Genome Using Histone Mark ChIP-Seq

**DOI:** 10.3390/genes11010003

**Published:** 2019-12-18

**Authors:** N. B. Kingsley, Colin Kern, Catherine Creppe, Erin N. Hales, Huaijun Zhou, T. S. Kalbfleisch, James N. MacLeod, Jessica L. Petersen, Carrie J. Finno, Rebecca R. Bellone

**Affiliations:** 1 Veterinary Genetics Laboratory, School of Veterinary Medicine, University of California, Davis, CA 95616, USA; nbkingsley@ucdavis.edu; 2 Department of Population Health and Reproduction, School of Veterinary Medicine, University of California, Davis, CA 95616, USA; enburns@ucdavis.edu (E.N.H.); cjfinno@ucdavis.edu (C.J.F.); 3 Department of Animal Science, University of California, Davis, CA 95616, USA; ckern@ucdavis.edu (C.K.); hzhou@ucdavis.edu (H.Z.); 4 Epigenetics Services Team, Diagenode, Liège 4102, Belgium; catherine.creppe@diagenode.com; 5 Gluck Equine Research Center, Department of Veterinary Science, University of Kentucky, Lexington, KY 40506, USA; ted.kalbfleisch@uky.edu (T.S.K.); jnmacleod@uky.edu (J.N.M.); 6 Department of Animal Science, University of Nebraska, Lincoln, NE 68588, USA; jessica.petersen@unl.edu

**Keywords:** FAANG, epigenetics, horse, genome regulation, H3K4me1, H3K4me3, H3K27ac, H3K27me3, tissue-specific, annotation

## Abstract

One of the primary aims of the Functional Annotation of ANimal Genomes (FAANG) initiative is to characterize tissue-specific regulation within animal genomes. To this end, we used chromatin immunoprecipitation followed by sequencing (ChIP-Seq) to map four histone modifications (H3K4me1, H3K4me3, H3K27ac, and H3K27me3) in eight prioritized tissues collected as part of the FAANG equine biobank from two thoroughbred mares. Data were generated according to optimized experimental parameters developed during quality control testing. To ensure that we obtained sufficient ChIP and successful peak-calling, data and peak-calls were assessed using six quality metrics, replicate comparisons, and site-specific evaluations. Tissue specificity was explored by identifying binding motifs within unique active regions, and motifs were further characterized by gene ontology (GO) and protein–protein interaction analyses. The histone marks identified in this study represent some of the first resources for tissue-specific regulation within the equine genome. As such, these publicly available annotation data can be used to advance equine studies investigating health, performance, reproduction, and other traits of economic interest in the horse.

## 1. Introduction

In 1992, researchers discovered the first disease mutation in horses, conferring hyperkalemic periodic paralysis (HYPP) in Quarter Horses [1], yet identification of additional equine genetic diseases progressed slowly, with only nine disease-associated variants discovered prior to 2007 [2,3]. Since the release of the equine reference genome in 2007 [4], 22 additional genes were found to cause or be associated with equine diseases, yet there are at least 200 described genetic disorders for which causal variants are unknown [2,5]. While the majority of characterized equine disease variants are located within coding regions, an increasing amount of research in humans and other animal species suggests that a large number of disease mutations are harbored within regulatory elements and other functional but non-coding regions of the genome [6,7,8,9]. Current genomic annotations for the horse have limited information about the functions of these non-coding regions, making it difficult to identify variants that alter gene regulation. While there is a high degree of conservation within coding regions across species, increasing evidence suggests that regulatory regions, especially tissue-specific elements, are not as well conserved in terms of sequence or function across species [7,8,9,10].

Using improved annotations of regulatory networks for humans and other mammals, researchers have begun to identify causal variants outside of coding regions. For example, a super-enhancer that significantly contributes to the risk of type II diabetes in humans was identified by combining a genome-wide association study (GWAS) with locations of regulatory elements and other functional regions across the genome [11]. Additionally, cattle researchers used annotations from both genomic assays on bovine tissue and homology-based methods to investigate complex traits. They found that the annotation from the homology-based method provided less relevant information than the bovine-specific annotation when combined with GWAS pertaining to milk production [8]. Along with the increasing number of regulatory GWAS, researchers are also expanding efforts to look at large-scale changes in the epigenome, such as associations between genome-wide changes in active regulatory elements and autism spectrum disorder [12]. With the increasing focus on the importance of regulatory elements in the pathogenesis of many diseases, it is clear that annotations of the equine genome need to encompass both coding and non-coding functional elements in order to understand complex genetic diseases and other traits affecting horses and the equine industry.

Similar to the Encyclopedia of DNA Elements (ENCODE), the Functional Annotation of ANimal Genomes (FAANG) initiative was established to improve the reference annotation of animal species, including characterization of genomic regulatory regions [13,14,15]. The link between chromatin modifications and regulatory regions, such as enhancers and promoters, has been well established for several decades [16]. Four histone tail modifications, also known as histone marks, were selected by the FAANG consortium to characterize promoters (H3K4me3) and enhancers (H3K4me1), as well as distinguish between active (H3K27ac) and inactive (H3K27me3) genomic elements [15].

Histone tail modifications were first hypothesized to affect genomic regulation in 1964 [17]. Since that time, associations between histone marks and regulatory elements have continued to expand. However, it is still unclear if the associations are the result of underlying functional roles in all cases or if the histone marks are the by-products of regulatory activities [18]. There is evidence that monomethylation of H3K4 recruits chromatin-modifying proteins to enhancer regions leading to enhancer priming and recognition [19]. Conversely, H3K4me3 is associated with promoters and the transcription start site, but some suggest that the mark may actually be a signature left by frequent transcriptional activity to create cellular memory rather than acting as a pioneer factor [18]. In conjunction with the H3K4 modifications, acetylation of H3K27 is found at active enhancers and promoters [20]. Trimethylation of the same residue, however, is strongly associated with facultative heterochromatin, leading to inactive regulatory elements and silenced genes [20]. In fact, these two H3K27 marks are thought to be antagonistic to one another, such that acetylation may actually prevent Polycomb silencing [21]. Acetylation of H3K27 also decreases the overall positive charge of the histone proteins, leading to fewer interactions with DNA and more open chromatin structure [22]. Since H3K27ac has been strongly associated with active regulatory elements across the genome, presence of this mark can be used to distinguish between active and poised regulatory elements [23]. While research into the functional roles of histone tail modifications continues, the associations between these four marks and patterns of regulatory elements are well established. For more than a decade, chromatin immunoprecipitation (ChIP) assays have remained the primary method for identifying genomic sites enriched with histone marks to conduct large-scale regulatory mapping [24,25].

As part of the international FAANG collaboration, we performed ChIP sequencing (ChIP-Seq) on four major histone modifications (H3K4me1, H3K4me3, H3K27ac, and H3K27me3) in eight equine tissues (adipose, brain (parietal cortex), heart, lamina, liver, lung, (skeletal) muscle, and ovary). These tissues were collected as part of the FAANG equine biobank, which includes samples from 80 tissues, six fluids, and two cell lines that were collected from two healthy adult thoroughbred mares [26]. The eight tissues evaluated in this study were prioritized for thorough investigation based on (1) continuity with other FAANG species to enable across species comparisons and/or (2) the primary needs of the equine community in terms of health, performance, and reproduction.

## 2. Materials and Methods

Due to the limited number of previous histone ChIP-Seq experiments across equine tissues, we first performed quality control (QC) testing to determine appropriate experimental parameters for each tissue and mark. Sequencing, rather than targeted qPCR, was used to assess the quality of the QC data due to the limited knowledge of appropriate tissue-specific control genes in the horse. All ChIP-Seq experiments, including QC, were conducted by Diagenode ChIP-Seq Profiling Service (Diagenode, Cat# G02010000, Liège, Belgium), and a complete summary of the final protocols used for all tissues can be accessed at ftp://ftp.faang.ebi.ac.uk/ftp/protocols/assays/. Quality control involved chromatin extraction, ChIP, library preparation, and sequencing of one training sample from each of the eight tissues investigated. Quality control training samples were obtained from previously banked tissues collected at the University of California, Davis. Bioinformatic analysis was performed on the QC data in order to calculate library complexity and ChIP enrichment metrics and to call peaks to evaluate the genomic distribution of detected marks. Library complexity metrics included non-redundant fraction (NRF) and PCR bottleneck coefficients 1 and 2 (PBC1 and PBC2). Metrics for ChIP enrichment included normalized strand cross-correlation coefficient (NSC), relative strand cross-correlation coefficient (RSC), and Jensen–Shannon distance (JSD). Non-redundant fraction, PBC1 and 2, NSC, and RSC are all standardized metrics of the ENCODE project [27] and were compared against ENCODE standards to determine the efficacy of the ChIP protocols. Jensen–Shannon distance is a common statistic used to compare two distributions that can be applied to ChIP datasets using deepTools version 2.4.3 [28], and a threshold was determined by agreement among FAANG collaborators. Tissues of interest (TOI) used in the final experiments that generated combined peak-calls were collected from two thoroughbred mares (referred to in this manuscript as ECA_UCD_AH1 for SAMEA104728862 and ECA_UCD_AH2 for SAMEA104728877) as part of the FAANG equine biobank [26], and all protocols for this work were approved by the University of California, Davis Institutional Animal Care and Use Committee (Protocol #19037). Laboratory procedures that varied based on tissue are summarized in Table 1.

### 2.1. Chromatin Extraction

Chromatin was extracted from adipose using the True MicroChIP Kit (Diagenode, Cat# C01010130, Liège, Belgium) and from the other seven tissues following the iDeal ChIP-Seq Kit for histones (Diagenode, Cat# C01010059, Liège, Belgium) with the modifications or specifications described in this paper. The starting amount varied depending on tissue, such that those with extensive extracellular matrices and/or low ratio of nuclei to cellular matter required larger amounts of starting material compared to those tissues that homogenized easily. Samples were homogenized either by douncing (liver) or grinding with the Tissue Lyser II (Qiagen, Germany) at 25 strokes/minute for a length of time that varied by tissue (Table 1).

In order to reach the desired fragment length (approximately 200 bp), chromatin was sheared with the Bioruptor Pico (Diagenode, Cat# B01060001, Liège, Belgium) combined with the Bioruptor^®^ Water cooler for 8–12 cycles of 30 s with 30 s rest between cycles. The temperature was maintained at 10 °C for adipose and 4 °C for all other tissues during shearing. The number of cycles varied based on tissue (Table 1), and the chromatin quality was assessed using the Fragment Analyzer (Aligent, Santa Clara, CA, USA).

### 2.2. Immunoprecipitation

Immunoprecipitation (IP) of the four histone marks, along with a negative IP control (IgG), was performed on tissue-specific amounts of chromatin using the IP-Star Compact Automated System (Diagenode, Cat# B03000002, Liège, Belgium). The antibodies used were all previously validated by Diagenode, and antibody concentrations were determined during QC for every tissue and histone mark combination (Table S1). An aliquot of chromatin from each tissue was set aside for the input to characterize sequencing background and identify true ChIP enrichment.

### 2.3. Sequencing

Libraries were prepared using the IP-Star^®^ Compact Automated System (Diagenode, Cat# B03000002, Liège, Belgium) and the MicroPlex Library Preparation Kit v2 (Diagenode, Cat# C05010013, Liège, Belgium) for the input and four ChIPs per tissue. Libraries were amplified prior to sequencing for at least 10 cycles, and additional cycles were performed as needed to reach a concentration of 3–10 nM. Using Agencourt AMPure XP (Bechman Coulter, Brea, CA, USA), libraries were purified and fragments were size-selected for approximately 200 bp. Libraries were sequenced as 50 bp, single-end reads on the HiSeq 4000 platform (Illumina, San Diego, CA, USA) to generate approximately 55–80 M raw reads for H3K27me3 and 30–50 M raw reads for the other marks and inputs. 

### 2.4. Data Processing

A complete summary of the bioinformatic workflow can be accessed at ftp://ftp.faang.ebi.ac.uk/ftp/protocols/analysis/, and bioinformatic parameters that varied by mark are summarized in Table 2. Reads were trimmed using Trim-Galore version 0.4.0 [29,30] under the default parameters and aligned to EquCab3.0 [31] with BWA-MEM version 0.7.9a [32], such that split hits were marked as secondary alignments. Alignments were converted to BAM file format, processed, and filtered using SAMtools version 1.9 [33]. For strict quality filtering, reads were removed if they did not map, had secondary alignments, failed platform/vendor quality tests, were identified as optical duplicates, or had an alignment quality score lower than 30. PCR duplicates were marked with PicardTools version 2.7.1 [34] and then removed with SAMtools. For peak-calling, MACS2 version 2.1.1.20160309 [35] was used to call peaks for all marks, and SICERpy version 0.1.1 [36], which is a wrapper for executing SICER [37], was also used to call peaks for the broad mark, H3K27me3. Combining peak-calls involved identifying overlapping regions of enrichment in both biological replicates where at least one replicate was significantly enriched based on a set of significance thresholds that varied by mark (Table 2). Additionally, enrichment tracks (bigWig files) were generated using deepTools version 2.4.3, which subtracted background characterized by the input and then combined enrichment from both replicates. 

### 2.5. Data Analysis

As with the QC data, the datasets from the eight TOI were assessed by calculating library complexity and ChIP enrichment metrics, as well as evaluating the genomic distribution of detected marks. Identity between peaks called for ECA_UCD_AH1 and ECA_UCD_AH2 were compared to assess the similarity of the biological replicates using the Jaccard Index [38], also known as the Jaccard Similarity Coefficient, from BEDtools version 2.27.1 [39]. Unique peaks were defined as a peak for a given mark that does not have any overlap with peaks from the same mark in the other prioritized tissues. BEDtools version 2.27.1 was used to identify unique peaks, as well as calculate the percent of the genome covered by peaks. Graphs were generated using ggplot2 with R software version 3.4.3 [40,41]. To characterize the average peak topology in relation to gene annotations and calculate normalized enrichment patterns, we used deepTools version 2.4.3. RNA-Seq data from the two FAANG replicates allowed for site-specific validation of the histone peaks (ERR2584116, ERR2584168, ERR2584153, ERR2584205, ERR2584194, ERR2584142, ERR2584135, ERR2584187, ERR2584195, ERR2584143, ERR2584197, ERR2584145, ERR2584144, ERR2584196, ERR2584152, and ERR2584204). Analysis of Motif Enrichment (AME) from the MEME Suite version 5.0.5 [42] was used to identify known transcription factor binding motifs within peaks based on the JASPAR 2018 vertebrate database [43], and Biological Process Gene Ontologies (GOs) from Swiss Prot were used to perform a GO term analysis [44]. Novel motifs were characterized with DREME and MEME, and each of the novel motifs was manually investigated to identify additional known motifs that were not included in the JASPAR database. The Integrated Genome Viewer [45] was used to visualize peak-calls in conjunction with the Ensembl annotation (release 95) for the EquCab3.0 reference assembly [46]. String version 11.0 was used to perform a protein–protein interaction analysis on the transcription factors implicated in each tissue based on the enriched motifs identified in the tissue-specific active enhancer elements [47]. 

## 3. Results

Quality control testing was performed to determine tissue-specific laboratory parameters such as antibody concentration and shearing time (Table 1 and Table S1) by comparing the quality metrics and peak-calls to ENCODE and FAANG standards (Table S2). The raw and processed data are available on https://data.faang.org/home under the study accession PRJEB35307. The processed files include read alignments to EquCab3.0 and peak-calls for each biological replicate, as well as the combined peak-calls.

### 3.1. Assessing Data Quality

**Figure 1:**
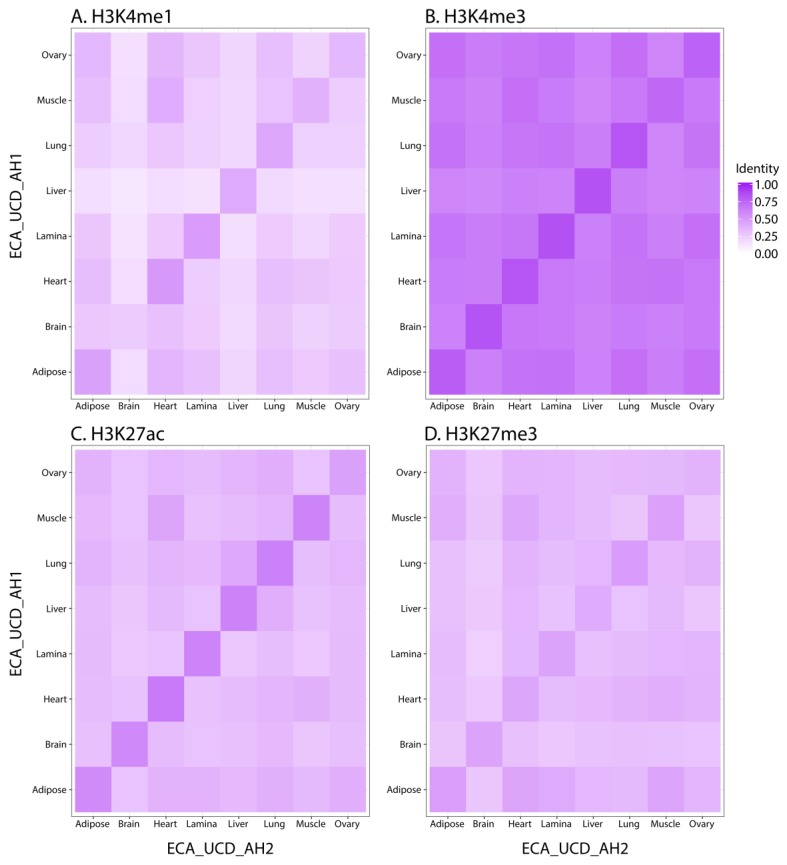
Jaccard Index to measure similarity of peaks called for each histone mark and tissue between biological replicates, ECA_UCD_AH1 and ECA_UCD_AH2. Each panel is a heatmap displaying the Jaccard Index for pairwise comparisons of tissues between replicates. Darker purple indicates that there are more peaks that are shared by the two tissues. (**A**) H3K4me1, (**B**) H3K4me3, (**C**) H3K27ac, (**D**) H3K27me3 called by SICER.

Using the Jaccard Index to compare replicates for all of the marks, we found the highest identity between the two replicates of the same tissue (Figure 1), with the exception of the brain replicates for H3K4me1 and the ovary replicates for H3K27me3. For the brain replicates, ECA_UCD_AH2 had 65,327 peaks compared to 143,328 peaks for ECA_UCD_AH1 (Table 3). In addition to a lower peak number, two of the library complexity quality scores for the ECA_UCD_AH2 brain replicate were lower than the ENCODE quality thresholds (Table 4). In terms of enrichment, however, the cross-correlation metrics for this sample were both above the established thresholds and indicate that the data had sufficient ChIP enrichment. Indeed, we were able to call 95,918 combined peaks, which is consistent with the range of H3K4me1 values from the other tissues. For the ovary replicates, two of the three library complexity metrics were below ENCODE standards, yet all of the ChIP enrichment metrics were consistent with those for the broad mark in other tissues, indicating that we had sufficient ChIP enrichment despite lower library complexity. When comparing SICER and MACS2 peak-calls for the broad mark, the number of combined peaks (8479 and 40,825, respectively) and the percentage of the genome covered (2.1% and 1.2%, respectively) for ovary were all consistent with the same measures for the H3K27me3 peaks from the other tissues evaluated in this study. 

While demonstrating more variation than the other marks, H3K27me3 had peaks called by SICER that were more similar between replicates of the same tissue compared to the H3K27me3 peaks called by MACS2 software (Figure S1). Peak-calls from the two biological replicates were combined by identifying regions of overlapping enrichment in which at least one replicate had significant enrichment based on a q-value that differed by mark (Table 2). For H3K4me1, H3K4me3, and H3K27ac, the number of combined peaks ranged from 93–121 K, 26–29 K, and 64–88 K, respectively. The number of combined peaks called for the broad mark was lower than the three activating marks for both MACS2 and SICER (24–68 K and 7–11 K, respectively). Although the combined peak numbers for the MACS2-H3K27me3 datasets were more similar to the ENCODE equivalent than the number of peaks called by SICER (Table S3), the SICER-H3K27me3 combined peaks covered a larger portion of the genome, similar to that for the other marks (Table 3). Files for H3K27me3 peaks called by MACS2 and SICER are both publicly available for every tissue. 

**Figure 2:**
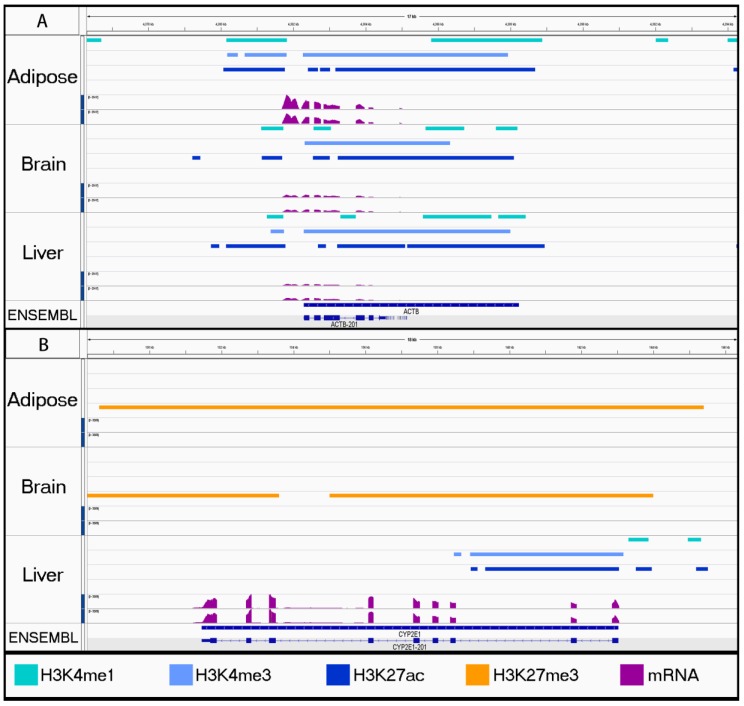
Proof-of-principle investigating house-keeping gene, *ACTB*, and liver-specific gene, *CYP2E1*, for appropriate regulatory elements. For adipose, brain, and liver tissue, combined peaks are displayed for H3K4me1 (aqua), H3K4me3 (light blue), H3K27ac (dark blue), H3K27me3 from SICER (orange), and mRNA expression (purple). (**A**) *ACTB* is a housekeeping gene that is commonly expressed for many tissues. (**B**) *CYP2E1* is a liver enzyme, which displays tissue-specific expression. Note the presence of the H3K27me3 repressive mark (orange) within adipose and brain samples.

As a proof-of-principle, we investigated a small number of regions near well-characterized genes to compare the histone peaks with RNA-seq data generated from the same tissues. We found consistent activating marks across all tissues for a widely expressed gene, *ACTB* (Figure 2A). Conversely, liver was the only tissue enriched for a set of active histone marks near the transcription start site (TSS) of the liver-specific gene *CYP2E1* (Figure 2B) [48]. 

### 3.2. Characterizing Tissue-Specific Features

**Figure 3:**
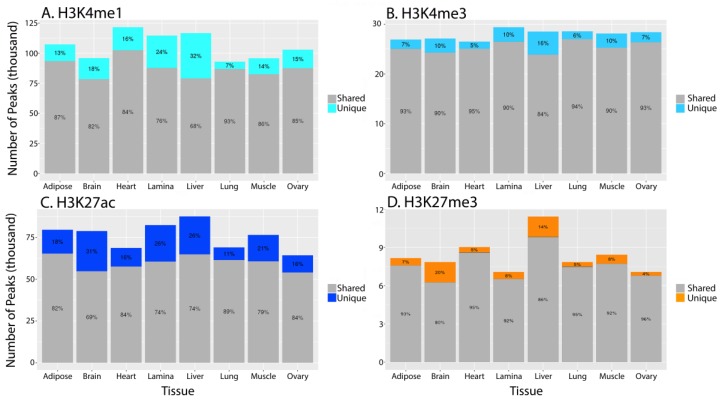
Tissue-specific peaks for each histone mark. Grey area indicates the number of peaks for a particular histone tail modification that are shared between at least two tissues, while the color region of each bar indicates the number of tissue-specific peaks. Percentage values are also assigned to the two segments of each bar to indicate the proportion of shared and unique peaks. (**A**) H3K4me1, (**B**) H3K4me3, (**C**) H3K27ac, (**D**) H3K27me3 from SICER.

Brain tissue had the highest percentage of unique peaks, defined as peaks that were only found in that tissue, for H3K27ac (31%) and H3K27me3 (20%), while liver had the highest percentage of unique peaks for H3K4me1 (32%) and H3K4me3 (16%), along with the second highest for H3K27ac (26%) and H3K27me3 (14%) (Figure 3). Lamina tissue also had a high percentage of unique peaks for the three activating marks with 24, 10, and 26% for H3K4me1, H3K4me3, and H3K27ac, respectively. 

**Figure 4:**
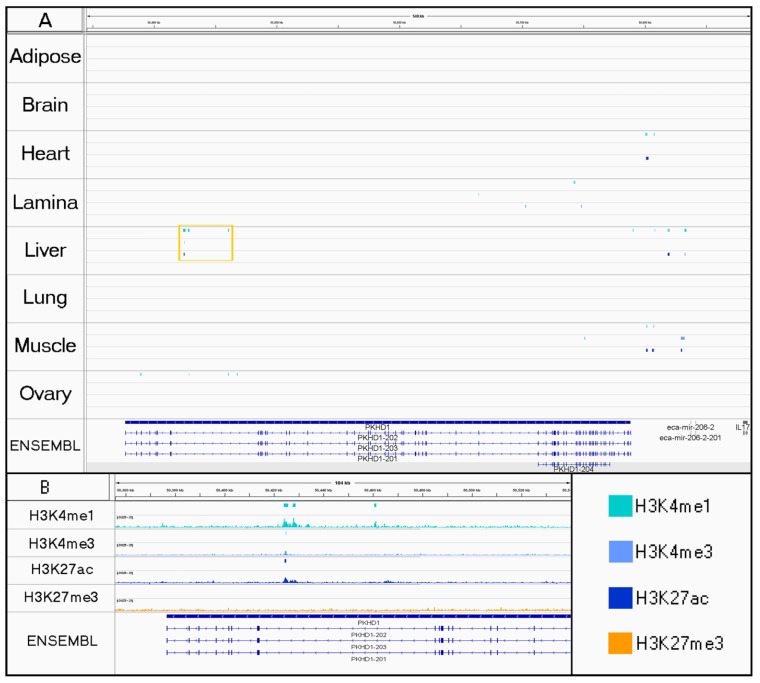
Evidence of a tissue-specific regulatory element found in liver tissue. For each tissue, peaks are displayed for H3K4me1 (aqua), H3K4me3 (light blue), H3K27ac (dark blue), and H3K27me3 from SICER (orange). (**A**) Gold box highlights liver-specific active marks in the 59th intron of an annotated gene, *PKHD1* (Ensembl Transcript ID: ENSECAT00000024985.1), which is transcribed from the antisense strand. H3K4me1 marks were also detected in ovary tissue at the end of the gene, but they do not indicate the presence of an active enhancer without co-occurrence of H3K27ac. (**B**) Enrichment profiles (BigWig) were visualized below the corresponding peak tracks for the region highlighted by the gold box in A.

**Figure 5:**
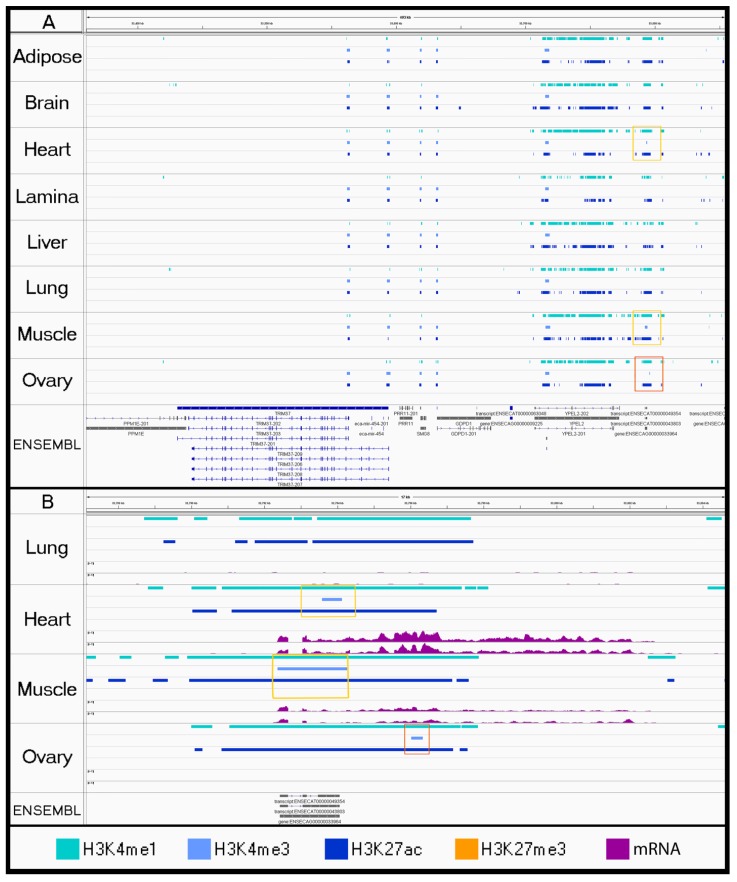
Visualizing tissue-specific peak-calls using the Integrated Genome Viewer. For each tissue, peaks are displayed for H3K4me1 (aqua), H3K4me3 (light blue), H3K27ac (dark blue), and H3K27me3 from SICER (orange). Gold boxes highlight active marks associated with promoters (H3K4me3) in both muscle tissues (skeletal and cardiac) for an unannotated ncRNA, *LOC111775680* (*ENSECAT00000049354*), and red box highlights an H3K4me3 peak specific to ovary tissue. (**B**) Zoomed in view of (**A**) for relevant tissues with RNA expression shown in purple. While ovary also appears to have a peak in H3K4me3 nearby, it does not have expression of the lncRNA based on mRNA expression from these tissue samples.

In addition to characterized genes, we also investigated a small number of genomic regions with putative tissue-specific functions in liver and muscle. For liver, a potential tissue-specific regulatory element was identified in the 59th intron of *PKHD1* (Ensembl Transcript ID: ENSECAT00000024985.1; Figure 4), a gene which has been previously associated with liver fibrosis [49]. Similarly, when considering a genomic region associated with racing ability [50], peaks for H3K4me3 in both muscle tissues were discovered at the start of a predicted lncRNA from Ensembl genebuild [51], indicating that this uncharacterized gene may be particularly informative for the function of contractile tissue (Figure 5).

**Figure 6:**
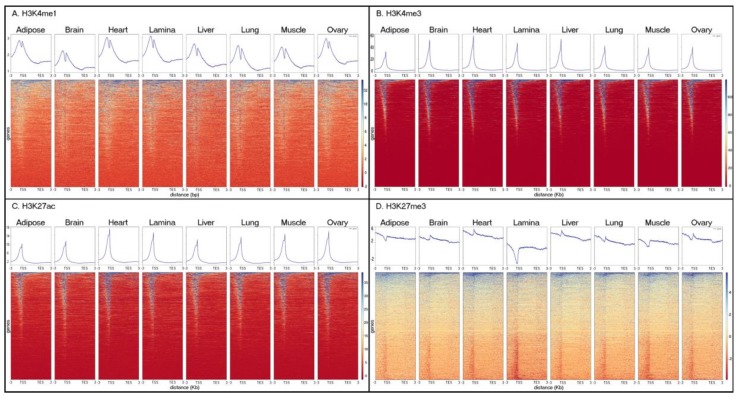
Histone mark enrichment across the average annotated gene body. (**A**) H3K4me1, (**B**) H3K4me3, (**C**) H3K27ac, (**D**) H3K27me3 from SICER. Topology plots (top) and heat maps (bottom) show average enrichment of each histone mark in each tissue across a size-normalized gene distribution based on the Ensembl (Release 95) annotation for EquCab3.0. Each line in the heatmap represents mark enrichment across a given gene, such that red indicates low relative enrichment and blue indicates high relative enrichment.

Across all tissues, H3K4me1 was enriched around the TSS, with a decrease in enrichment at the actual annotated start site that created a bimodal distribution across the average gene body (Figure 6A). Additionally, H3K4me1 maintained a moderate level of enrichment throughout the gene body, as well as 3 Kb up- and downstream, as expected for distal and proximal enhancer elements. Alternatively, H3K4me3 (Figure 6B) and H3K27ac (Figure 6C) had peaks of enrichment at the TSS, although H3K27ac also showed enrichment, to a lesser extent, just upstream of the TSS. H3K27me3 had lower enrichment than the other three marks overall, but the average enrichment was essentially constant throughout the gene body, as well as 3 Kb up- and downstream (Figure 6D). While patterns of enrichment for each mark were highly consistent between tissues, the enrichment of H3K27me3 for lamina was lower at the TSS compared to the other tissues. 

### 3.3. Identifying Motifs and Biological Process GO Terms

**Figure 7:**
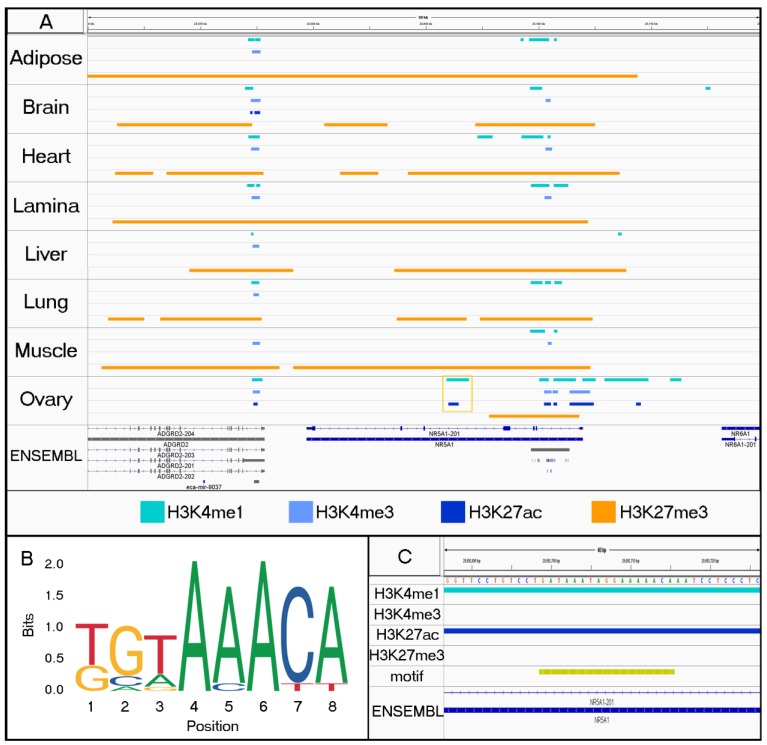
Localizing enriched TF binding motifs within tissue-specific peaks. For each tissue, peaks are displayed for H3K4me1 (aqua), H3K4me3 (light blue), H3K27ac (dark blue), and H3K27me3 from SICER (orange). Gold box highlights ovary specific marks in intron of *NR5A*. (**B**) Motif logo displays one of the major motifs for FOXO3. (**C**) Zoomed in view of 40 bp within region highlighted in (**A**). The gold track indicates the presence of two nearly consecutive motifs for FOXO3 within the H3K4me1 and H3K27ac peaks that were detected only in ovary tissue.

**Figure 8:**
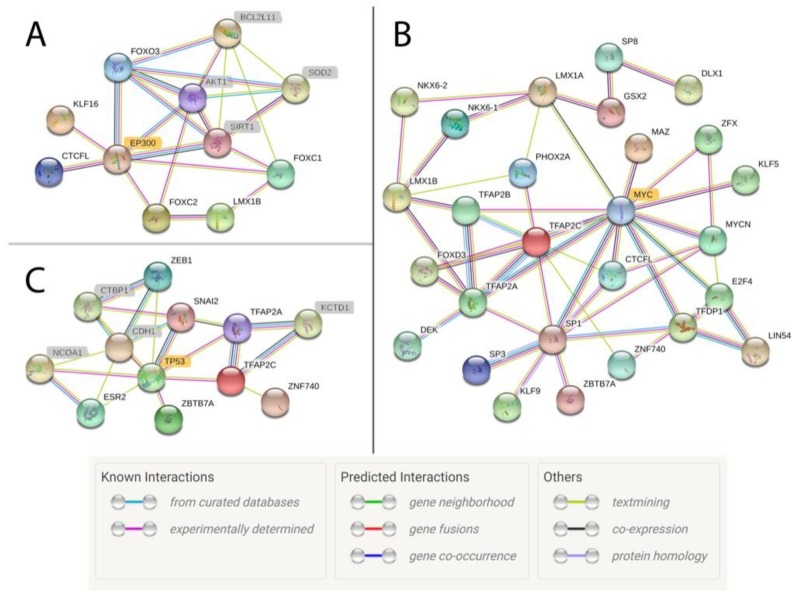
Network of transcription factor interactions based on active motif regions in ovary, skeletal muscle, and lung tissues. Each circle represents a protein, and lines indicate interactions between proteins. Each line color indicates a different type of evidence for the interaction. Protein names highlighted in grey were not identified in the horse ChIP-seq data but were identified as secondary factors by the network analysis based on implicated or known protein–protein interactions from research in humans and mice. (**A**) EP300 is highlighted in gold to denote its central role in the network for ovary tissue. (**B**) MYC is highlighted in gold to indicate its central role in the network for muscle tissue. (**C**) TP53 is highlighted in gold to indicate its central role in the network for lung. Legend is from String Software [47].

We identified between 16 and 61 transcription factor binding motifs in the unique active regions for each tissue, and the full results from the GO term analysis can be found in Tables S4–S19. While a large proportion of the identified transcription factors were still shared between tissues despite being identified in tissue-specific active regions, all of the tissues except for adipose and lung had at least one uniquely detected transcription factor binding site. A uniquely detected binding site was defined as a motif that was only identified in the tissue-specific active regions for a single tissue. The motifs were ranked based on significance of enrichment after multiple testing correction, and the top five enriched and identified motifs for each tissue are listed in Table 5. Ovary was the only tissue that had uniquely detected transcription factor motifs in this top five list, and the most significant motif was associated with FOXO3, which is a transcription factor (TF) characterized by 39 GO terms, including several for tissue-specific functions such as oocyte maturation (GO:0001556) and ovulation (GO:0001542). In fact, upon closer inspection, the FOXO3 motif was found within a tissue-specific regulatory element near a gene with recognized roles in mammalian reproduction, *NR5A1* (Figure 7) [52]. Using a network analysis for TFs implicated in each tissue, we found that six networks contained EP300 as a central node, although these networks did not link every TF for a given tissue (Figure 8A). Interestingly, MYC was the central node for brain, liver, and skeletal muscle (Figure 8B), and TP53 was the central node for lung (Figure 8C). 

## 4. Discussion

As part of the FAANG consortium, we mapped more than 1 million putative regulatory sites across the equine genome, which will contribute significantly to our understanding of genome regulation in horses, as well as regulatory differences across species. To ensure that we obtained high quality data for the horse, we first performed QC experiments to optimize tissue-specific laboratory protocols (Table S2). Adipose tissue presented a distinct challenge for chromatin extraction due to the low number of nuclei and large amount of cellular material, including lipid deposits. Efforts to obtain high quality data were successful, as quality metrics for this tissue surpassed ENCODE standards or scored within the range of the other tissues for a given mark. From this work, we suspect that other difficult tissues (i.e., those with low nuclei density, extensive and persistent extra- or intracellular material, etc.) will also require similar alternate approaches for chromatin extraction, such as additional starting material or specialized kits. 

To ensure accurate and relevant peak-calling, we employed MACS2 software for calling H3K4me1, H3K4me3, and H3K27ac peaks and both MACS2 and SICER for H3K27me3 peaks. While several attempts have been made to develop a bench-marking method for ranking ChIP peak-callers, there is no established gold standard for selecting a particular application [53]. Steinhauser et al., for example, found that SICER was better able to detect true-positive differential regions (DRs) when H3K36me3 data were down-sampled to as low as 10%, while MACS2 was only effective at detecting true-positive DRs at 60% or higher [54]. SICER, however, had a higher number of false-positive DRs compared to MACS2, indicating that it may be sacrificing some specificity to obtain higher sensitivity, while MACS2 does the opposite. Based upon generating peaks with higher identity between replicates (Figure S1) and higher proportions of the genome covered (Table 3), we found SICER software to be more consistent for calling broad peak-calls compared to MACS2, which is consistent with previous research [55]. For that reason, we continued our investigations using the SICER-called H3K27me3 peaks. Given that peaks from both callers are supported in the literature for other species studied, both sets of peak-calls are available at https://data.faang.org/dataset/PRJEB35307. 

Using the Jaccard Similarity Index to compare significant peak-calls, we determined that biological replicates were highly similar for each tissue with the exception of the brain replicates for H3K4me1 and the ovary replicates for H3K27me3 (Figure 1). While these low identity scores could be the result of underlying biological differences between the samples of brain or ovary tissue, this is unlikely given that the differences between replicates were only found in one mark for each of the two tissues. Using a bioinformatic method that relied on identifying overlapping enrichment rather than the intersection of significant peaks ensured accurate combined peak calling despite lower similarity scores. Combined peak-calls were assessed for significant enrichment surrounding genes with known expression patterns. In particular, the three active marks (H3K4me1, H3K4me3, and H3K27ac) had consistent peaks between tissues near *ACTB* (Figure 2A) and tissue-specific peaks near the liver enzyme *CYP2E1* (Figure 2B), which support the validity of our peak-calling methods. Additionally, these peak-calls were all consistent with RNA expression from the same tissues for ECA_UCD_AH1 and ECA_UCD_AH2, further supporting the validity of the enrichment patterns detected for each histone mark.

Comparing replicated peaks between tissues, we found that liver, brain, and lamina had the highest percentage of peaks that were unique to only that tissue (Figure 3). Liver is known to have hundreds of distinct biological functions [48]. Since many of the functions are entirely unique to liver, a high proportion of unique active regulatory elements is consistent with the specialization of this tissue. Additionally, the mammalian brain is thought to have hundreds of different neuronal cell types within the cortex to coordinate numerous neurologic functions simultaneously, making it one of the most complex tissues of the body [56]. Therefore, these results are consistent with the expectation that there is a high degree of regulatory specificity to control numerous coordinated functions within complex tissues. 

Interestingly, lamina was the other tissue with a high percentage of unique peaks for H3K4me1, H3K4me3, and H3K27ac (Figure 3). Due to the role of lamina in disease, this finding may be particularly impactful for research into the physiological changes associated with laminitis, a syndrome that was established as a priority for equine research by the American Association of Equine Practitioners [57,58]. Additionally, when comparing the distribution of marks across the average gene body, we found that lamina tissue had an unusually low level of the repressive mark, H3K27me3, at the TSS (Figure 6). While all of the tissues had a portion of genes that appear to have a dip in enrichment directly at the TSS, lamina had the most extreme decrease. Perhaps the dip in enrichment for lamina is an indication of increased levels of expression across more of the genome and, when combined with the high percentage of unique peaks, could suggest that hoof lamina may perform additional biological processes that are currently uncharacterized. In order to understand all of the molecular functions of lamina tissue and its role in laminitis, more work is needed to further annotate and functionally characterize the extent of the cellular processes within healthy and diseased tissue. Without further validation, however, we cannot exclude that this decrease in enrichment may be a technical artifact such as decreased detection of H3K27me3 in lamina tissue due to low cell numbers and excess extracellular matrix. 

To further characterize tissue-specific regulatory regions, we identified numerous transcription factor binding motifs within the unique active elements for each tissue (Table 5). Despite investigating elements that were only enriched in one tissue, we still identified many transcription factor motifs that were shared between tissues (Table 4 and Table S19) and, based on a GO term analysis, many of these factors had numerous associated biological processes (Tables S4–S18). Our network analysis identified EP300 as a central secondary factor needed to connect many implicated TFs (Figure 8A). This gene encodes a histone acetyltransferase that is known to interact with many TFs by protein–protein interactions rather than DNA binding [59,60]. Given that the TF motifs were all identified within active elements based on the presence of H3K27ac, finding a connection with EP300 supports our ability to detect relevant peaks for this histone modification. Interestingly, there were three tissues (brain, liver, and muscle) that had MYC rather than EP300 as the central node for their TF networks (Figure 8B). We found that binding motifs for MYC were enriched in all three tissues, which is consistent with its role as a TF for many common cellular processes, including growth and regulation of the cell cycle [61]. Similarly, TP53 was the central node in the protein interaction network for lung tissue (Figure 8C), which is consistent with its role as a key regulator of the cell cycle when functional and as a major tumorigenesis factor in lung cancer when dysfunctional [62]. 

By developing tissue-specific maps of the H3K4me1, H3K4me3, H3K27ac, and H3K27me3 histone modifications, we aimed to build upon our current knowledge about genomic regulation in the horse and provide new resources for further advancing research on health, performance, and reproduction traits. In particular, incorporating histone ChIP-seq data with current annotations is expected to lead to the discovery of more tissue-specific functional variants that are outside of protein-coding regions. For example, a genome-wide investigation of liver fibrosis in the Swiss Franches-Montagnes breed led to the identification of an associated region containing a promising candidate gene, *PKHD1*, which was also associated with kidney and liver disease in humans [49]. Sequencing the coding region of this gene identified two synonymous coding variants strongly associated with disease; however, their causal role remains unclear. Comparing peaks from multiple tissues in that region, there are also several liver-specific regulatory peaks within intron 59 of *PKHD1* (Ensembl Transcript ID: ENSECAT00000024985.1) that may affect expression of this or another hepatic gene nearby (Figure 4) and represent another avenue to explore for the molecular mechanism underlying liver fibrosis. 

Tissue-specific histone modifications have also been implicated in the complex traits of performance [63] and, throughout modern horse domestication, performance abilities such as speed have been a major factor for selective breeding [64]. For example, recent research comparing three trotting breeds in Scandinavia (North Swedish Draught, Norwegian–Swedish Coldblooded trotter, and Standardbred trotter) found a 684 Kb genomic region associated with trotting racing ability containing numerous annotated and unannotated genes [50]. Using a protein-coding annotation, only one gene in the region, *TRIM37*, was identified as a potential candidate due to its associations with growth phenotypes in humans. Utilizing the locations of histone mark enrichment could help to identify other candidates such as tissue-specific regulatory regions or the genes that they regulate. In particular, there are H3K4me3 peaks at the start of a novel lncRNA predicted by Ensembl genebuild [51] within the region of interest for racing ability (Figure 5A). While uncharacterized, *LOC111775680* has a set of unique promoters found in skeletal and cardiac muscle with corresponding transcript data (as determined by RNAseq of the same tissues from ECA_UCD_AH1 and ECA_UCD_AH2), suggesting that the lncRNA may play a role in muscle physiology (Figure 5B). Additional work is needed to further characterize *LOC111775680* and investigate its potential role in muscle tissue and any corresponding effects on racing ability.

In addition to health and performance, these annotation data can also be used to identify important genomic regulatory regions for the reproductive system. Previously, homology with humans and mice was utilized to create a panel of candidate regions for determining the genetic cause of gonadal dysgenesis disorders across many breeds of horses [65], yet the panel was focused on coding variants and those within untranslated regions that may affect RNA and protein synthesis. By evaluating histone marks, we found that there are additional regulatory regions relevant to the reproductive system that were missed by the homology-based approaches. For example, *NR5A1*, a gene that is implicated in many dysgenesis cases [52,66], has a regulatory region as identified by H3K4me1 and H3K27ac histone modifications in equine ovary tissue that is characteristic of an active enhancer (Figure 7A). Upon further inspection, we identified two FOXO3 binding sites within the region enriched with peak-calls (Figure 7B,C). FOXO3 is a transcription factor with known roles in oocyte maturation [GO:0001556] and ovulation [GO:0001542] that was identified as an ovary-specific TF based on our motif analysis (Table S18). 

Along with the regulatory regions identified in this study, the equine FAANG consortium is characterizing the full RNA profile of more than 50 tissues and cell types from the FAANG equine biobank supported by a research community initiative [26] and the DNA methylation profiles for the TOI. Moreover, the equine FAANG consortium is currently mapping a major insulator protein known as CTCF, with the goal of generating tissue-specific chromatin state predictions for the eight TOI. CTCF plays a key role in defining chromatin looping and, therefore, topologically associated domains and gene-enhancer interactions when combined with histone ChIP-Seq [67,68]. The same panel of genomic investigations are also being conducted in two stallions, so that males and females are represented in the annotations. We anticipate that the integration and utilization of these functional annotation datasets by the equine genomics community will lead to the identification of causal, non-coding variants underlying many traits of interest for equine medicine, performance, and reproduction. 

## Figures and Tables

**Table 1:** Optimized ChIP-Seq experimental procedures for each tissue.

Parameter	Adipose	Brain	Heart	Lamina	Liver	Lung	Muscle	Ovary
Starting Tissue (mg)	220	90	105	100	40	40	100	85
Homogenization Time (min)	8	5	5	9	n/a	5	5	5
Duration Fixed (min)	10	9	9	9	9	9	9	9
Fixation Temp. (°C)	37	23	23	23	23	23	23	23
Shearing Volume (uL)	400	1500	1800	1800	1500	1500	1800	1800
Shearing Cycles	5 × 8 ^1^	10	13	10	12	10	12	10
Chromatin per IP (ng)	700	300	500	700	450	800	260	1200

^1^ Samples were sonicated for five sets of eight cycles.

**Table 2:** Software parameters used to analyze ChIP-Seq data for each histone mark.

Software	Parameter	H3K4me1	H3K4me3	H3K27ac	H3K27me3
**MACS2**	Filtering	strict	strict	strict	strict
	Size	narrow ^1^	narrow	narrow	broad
	Size Flag	none	none	none	--broad
	Model	--fix-bimodal	--fix-bimodal	--fix-bimodal	--fix-bimodal
	Genome Size	2,409,143,234	2,409,143,234	2,409,143,234	2,409,143,234
**both**	Fragment Size	200	200	200	200
	FDR	0.05	0.01	0.01	0.1
**SICERpy ^2^**	Gap Size	n/a	n/a	n/a	4
	Window Size	n/a	n/a	n/a	200
	Genome Fraction	n/a	n/a	n/a	0.63

^1^ Mark was treated as broader than other narrow marks due to being categorized previously as broad by ENCODE [19]. ^2^ SICERpy was only used to call peaks for the broad mark, H3K27me3.

**Table 3:** Summary of peak number and percent of the genome covered for each mark and tissue.

Mark	Tissue	Software	Combined Peak Number	Percent Genome Covered	AH1 Peak Number	AH2 Peak Number
**H3K4me1**	Adipose	MACS2	107,318	5.1	130,242	157,497
	Brain	MACS2	95,918	3.1	143,328	65,327
	Heart	MACS2	121,663	4.9	137,385	155,881
	Lamina	MACS2	114,708	4.2	137,575	124,150
	Liver	MACS2	116,760	3.6	97,863	135,122
	Lung	MACS2	92,972	2.9	90,687	109,001
	Muscle	MACS2	95,816	3.7	137,322	100,999
	Ovary	MACS2	102,986	4.3	166,303	133,209
**H3K4me3**	Adipose	MACS2	26,905	1.7	26,286	29,121
	Brain	MACS2	27,101	1.6	25,473	28,028
	Heart	MACS2	26,475	1.4	24,101	27,985
	Lamina	MACS2	29,380	1.6	29,023	19,742
	Liver	MACS2	28,498	1.5	28,204	28,222
	Lung	MACS2	28,546	1.6	30,048	27,779
	Muscle	MACS2	28,110	1.6	30,428	25,123
	Ovary	MACS2	28,378	1.7	30,522	29,192
**H3K27ac**	Adipose	MACS2	79,620	3.3	75,823	99,249
	Brain	MACS2	78,823	3.2	89,445	73,795
	Heart	MACS2	68,728	2.9	71,462	7192
	Lamina	MACS2	82,394	2.9	91,345	78,953
	Liver	MACS2	87,589	3.1	84,814	96,238
	Lung	MACS2	69,054	2.9	69,621	75,299
	Muscle	MACS2	76,495	2.9	78,047	86,524
	Ovary	MACS2	64,318	3.3	94,817	82,318
**H3K27me3**	Adipose	MACS2	25,183	0.6	8948	29,906
	Brain	MACS2	24,243	0.6	16,055	23,411
	Heart	MACS2	68,113	1.8	31,455	88,818
	Lamina	MACS2	37,366	0.8	31,839	28,508
	Liver	MACS2	63,874	1.3	93,423	23,888
	Lung	MACS2	30,191	0.7	32,385	18,124
	Muscle	MACS2	42,610	0.9	39,076	29,579
	Ovary	MACS2	40,825	1.2	43,036	33,220
**H3K27me3**	Adipose	SICER	8167	4.9	13,540	14,571
	Brain	SICER	7860	3.4	11,386	13,603
	Heart	SICER	9032	3.3	12,192	18,903
	Lamina	SICER	7072	3.8	11,933	11,694
	Liver	SICER	11,430	3.7	22,270	16,099
	Lung	SICER	7863	2.6	12,668	11,715
	Muscle	SICER	8437	4.6	17,073	10,987
	Ovary	SICER	7083	3.0	14,731	11,124

**Table 4:** Quality metrics for assessing library complexity and ChIP enrichment. Thresholds for NRF, PBC1, PBC2, NSC, and RSC represent those developed by the Encyclopedia of DNA Elements (ENCODE) [27]. JSD threshold was established among members of the Functional Annotation of ANimal Genomes (FAANG) consortium.

Mark	Tissue	Rep	NRF	PBC1	PBC2	NSC	RSC	JSD
Thresholds			(>0.5)	(>0.5)	(>1)	(>1.05)	(>0.8)	(>0.05)
**H3K4me1**	Adipose	AH2	0.677	0.673	3.017	1.068	1.249	0.281
	Adipose	AH1	0.621	0.617	2.595	1.067	1.147	0.239
	Brain	AH2	0.435	0.443	1.908	1.055	1.243	0.186
	Brain	AH1	0.754	0.756	4.128	1.074	1.275	0.228
	Heart	AH2	0.708	0.708	3.444	1.086	1.790	0.321
	Heart	AH1	0.497	0.496	2.023	1.071	1.657	0.259
	Lamina	AH2	0.606	0.606	2.551	1.093	1.628	0.281
	Lamina	AH1	0.561	0.562	2.311	1.088	1.809	0.283
	Liver	AH2	0.760	0.762	4.226	1.097	1.240	0.226
	Liver	AH1	0.838	0.842	6.462	1.117	1.289	0.252
	Lung	AH2	0.736	0.736	3.796	1.079	1.123	0.199
	Lung	AH1	0.667	0.665	2.980	1.069	1.063	0.178
	Muscle	AH2	0.706	0.706	3.418	1.077	1.030	0.210
	Muscle	AH1	0.576	0.573	2.338	1.084	1.200	0.259
	Ovary	AH2	0.712	0.712	3.488	1.077	1.265	0.245
	Ovary	AH1	0.692	0.691	3.240	1.085	2.117	0.313
**H3K4me3**	Adipose	AH2	0.595	0.604	2.581	1.322	1.391	0.382
	Adipose	AH1	0.559	0.571	2.389	1.313	1.501	0.354
	Brain	AH2	0.497	0.515	2.167	1.366	1.198	0.516
	Brain	AH1	0.333	0.362	1.813	1.360	1.249	0.528
	Heart	AH2	0.410	0.435	1.905	1.467	1.364	0.540
	Heart	AH1	0.337	0.374	1.857	1.399	1.639	0.548
	Lamina	AH2	0.529	0.551	2.345	1.384	1.188	0.467
	Lamina	AH1	0.571	0.594	2.606	1.380	1.289	0.465
	Liver	AH2	0.452	0.471	1.996	1.407	1.196	0.517
	Liver	AH1	0.421	0.444	1.926	1.385	1.282	0.537
	Lung	AH2	0.610	0.628	2.813	1.354	1.154	0.387
	Lung	AH1	0.580	0.600	2.634	1.344	1.117	0.452
	Muscle	AH2	0.240	0.277	1.818	1.340	1.354	0.441
	Muscle	AH1	0.559	0.567	2.350	1.350	1.164	0.448
	Ovary	AH2	0.633	0.646	2.926	1.315	1.191	0.428
	Ovary	AH1	0.603	0.622	2.779	1.335	1.220	0.439
**H3K27ac**	Adipose	AH2	0.678	0.677	3.087	1.223	1.605	0.313
	Adipose	AH1	0.537	0.532	2.129	1.250	1.800	0.333
	Brain	AH2	0.495	0.493	2.001	1.202	1.320	0.310
	Brain	AH1	0.655	0.657	2.939	1.200	1.341	0.326
	Heart	AH2	0.493	0.489	1.970	1.316	2.193	0.402
	Heart	AH1	0.573	0.573	2.361	1.331	1.856	0.376
	Lamina	AH2	0.597	0.596	2.486	1.296	1.655	0.351
	Lamina	AH1	0.657	0.662	3.006	1.304	1.711	0.345
	Liver	AH2	0.719	0.722	3.651	1.258	1.225	0.347
	Liver	AH1	0.721	0.724	3.674	1.242	1.237	0.298
	Lung	AH2	0.500	0.499	2.008	1.241	1.290	0.327
	Lung	AH1	0.654	0.658	2.956	1.208	1.281	0.299
	Muscle	AH2	0.605	0.604	2.524	1.291	1.306	0.335
	Muscle	AH1	0.510	0.511	2.072	1.285	1.335	0.381
	Ovary	AH2	0.733	0.736	3.816	1.254	1.309	0.374
	Ovary	AH1	0.678	0.678	3.112	1.224	1.461	0.391
**H3K27me3**	Adipose	AH2	0.646	0.641	2.751	1.057	0.659	0.101
	Adipose	AH1	0.650	0.647	2.809	1.053	0.592	0.067
	Brain	AH2	0.511	0.510	2.077	1.060	0.400	0.101
	Brain	AH1	0.616	0.614	2.587	1.067	0.477	0.088
	Heart	AH2	0.407	0.414	1.834	1.070	0.595	0.106
	Heart	AH1	0.287	0.315	1.778	1.090	0.649	0.102
	Lamina	AH2	0.459	0.460	1.919	1.069	0.656	0.071
	Lamina	AH1	0.429	0.436	1.885	1.076	0.732	0.065
	Liver	AH2	0.545	0.537	2.140	1.076	0.648	0.093
	Liver	AH1	0.454	0.451	1.871	1.084	0.661	0.123
	Lung	AH2	0.619	0.615	2.575	1.072	0.617	0.072
	Lung	AH1	0.550	0.545	2.199	1.084	0.671	0.088
	Muscle	AH2	0.534	0.526	2.098	1.070	0.597	0.070
	Muscle	AH1	0.476	0.472	1.914	1.079	0.689	0.103
	Ovary	AH2	0.524	0.520	2.103	1.071	0.587	0.066
	Ovary	AH1	0.495	0.489	1.970	1.077	0.688	0.101

**Table 5:** Top five significantly enriched transcription factor binding motifs identified in tissue-specific active enhancers. Tissue specificity of the active enhancers was defined by overlap of H3K4me1 and H3K27ac in the same tissue and no overlap of these marks in this region in any other tissues, and tissue specificity of the binding motifs was then defined by detection of an enriched binding motif in only one tissue.

Rank	Motif ID	Consensus	Adjusted *p*-Value	UniProt Entry
**Adipose**				
1	SP3	VCCACGCCCMC	1.49 × 10^−10^	Q02447
2	TFDP1	VSGCGGGAAVN	1.74 × 10^−10^	Q14186
3	TFAP2A	HGCCYSAGGCD	3.27 × 10^−10^	P05549
4	TFAP2C	YGCCYBVRGGCA	4.56 × 10^−10^	Q92754
6	KLF16	GMCACGCCCCC	5.81 × 10^−9^	Q9BXK1
**Brain**				
1	TFAP2A(var.2)	YGCCCBVRGGCR	1.82 × 10^−16^	P05549
2	TFAP2B	YGCCCBVRGGCA	1.29 × 10^−13^	Q92481
3	SP3	VCCACGCCCMC	2.69 × 10^−13^	Q02447
4	TFAP2C	YGCCYBVRGGCA	4.99 × 10^−13^	Q92754
5	KLF16	GMCACGCCCCC	1.06 × 10^−12^	Q9BXK1
**Heart**				
1	MZF1	BGGGGA	2.23 × 10^−5^	P28698
2	Ascl2	ARCAGCTGCY	7.06 × 10^−4^	Q99929
3	ASCL1	VSAGCAGCTGSNN	9.41 × 10^−4^	P50553
4	SP3	VCCACGCCCMC	1.42 × 10^−3^	Q02447
5	NEUROD1	NRACAGATGGYNN	1.60 × 10^−3^	Q13562
**Lamina**				
1	SP2	GYCCCGCCYCYBSSS	8.51 × 10^−15^	Q02086
2	SP1	GCCCCKCCCCC	5.98 × 10^−14^	P08047
3	SP3	VCCACGCCCMC	2.84 × 10^−13^	Q02447
4	KLF16	GMCACGCCCCC	3.22 × 10^−13^	Q9BXK1
7	Zfx	SSSGCCBVGGCCTS	1.06 × 10^−11^	P17010
**Liver**				
1	SP1	GCCCCKCCCCC	7.81 × 10^−13^	P08047
2	TFAP2B	YGCCCBVRGGCA	4.23 × 10^−12^	Q92481
3	TFAP2C	YGCCYBVRGGCA	1.87 × 10^−11^	Q92754
4	TFAP2A	HGCCYSAGGCD	4.97 × 10^−11^	P05549
5	ZNF740	MCCCCCCCAC	8.99 × 10^−11^	Q8NDX6
**Lung**				
1	THAP1	YTGCCCDBA	5.09 × 10^−9^	Q9NVV9
3	ESR2	AGGTCASVNTGMCCY	1.08 × 10^−8^	Q92731
4	Zfx	SSSGCCBVGGCCTS	1.44 × 10^−8^	P17010
5	ZBTB7A	NVCCGGAAGTGSV	1.46 × 10^−8^	O95365
6	TFAP2A(var.2)	YGCCCBVRGGCR	6.51 × 10^−8^	P05549
**Muscle**				
1	SP1	GCCCCKCCCCC	1.45 × 10^−9^	P08047
2	SP2	GYCCCGCCYCYBSSS	4.21 × 10^−9^	Q02086
3	SP8	RCCACGCCCMCY	1.15 × 10^−8^	Q8IXZ3
4	CTCFL	CRSCAGGGGGCRSB	3.44 × 10^−8^	Q8NI51
5	KLF16	GMCACGCCCCC	4.36 × 10^−7^	Q9BXK1
**Ovary**				
1	FOXO3 ^1^	DAAAYA	7.23 × 10^−7^	O43524
3	KLF16	GMCACGCCCCC	1.93 × 10^−4^	Q9BXK1
4	FOXC1 ^1^	WAWGTAAAYAW	2.39 × 10^−4^	Q12948
6	CTCFL	CRSCAGGGGGCRSB	4.38 × 10^−4^	Q8NI51
7	Arid5a	SYAATATTGVDANH	4.99 × 10^−4^	Q03989

^1^ Transcription factor (TF) motifs that were only detected in one tissue.

## Supplementary Materials

The following are available online at https://www.mdpi.com/2073-4425/11/1/3/s1: Figure S1. Jaccard Index to measure similarity of peaks called for H3K27me3 peaks based on either MACS2 or SICER peak-calling; Table S1. Concentrations and catalog numbers for antibodies used during ChIP; Table S2. Quality metrics, peak-calling summary, and comparison to Encyclopedia of DNA Elements (ENCODE) data from quality control (QC) testing; Table S3. Comparing peak numbers to ENCODE data; Table S4. Motif and gene ontology (GO) term analysis results for adipose-specific active enhancers; Table S5. Motif and GO term analysis results for adipose-specific active promoters; Table S6. Motif and GO term analysis results for brain-specific active enhancers; Table S7. Motif and GO term analysis results for brain-specific active promoters; Table S8. Motif and GO term analysis results for heart-specific active enhancers; Table S9. Motif and GO term analysis results for heart-specific active promoters; Table S10. Motif and GO term analysis results for lamina-specific active enhancers; Table S11. Motif and GO term analysis results for lamina-specific active promoters; Table S12. Motif and GO term analysis results for liver-specific active enhancers; Table S13. Motif and GO term analysis results for liver-specific active promoters; Table S14. Motif and GO term analysis results for lung-specific active enhancers; Table S15. Motif and GO term analysis results for lung-specific active promoters; Table S16. Motif and GO term analysis results for muscle-specific active enhancers; Table S17. Motif and GO term analysis results for muscle-specific active promoters; Table S18. Motif and GO term analysis results for ovary-specific active enhancers; Table S19. Summary of the GO term analysis results for unique active elements from all tissues.
