# Supplementary material for: Functionally Annotating Regulatory Elements in the Equine Genome Using Histone Mark ChIP-Seq
Source: Genes (Basel). 2019 Dec 18;11(1):3. doi: 10.3390/genes11010003 (PMC7017286; doi:10.3390/genes11010003)
Supplement: Supplementary file 1 [file genes-11-00003-s001.zip › Supplement/Supplemental_Figures_and_Table_Descriptions.docx]

**Supplemental Figures:**


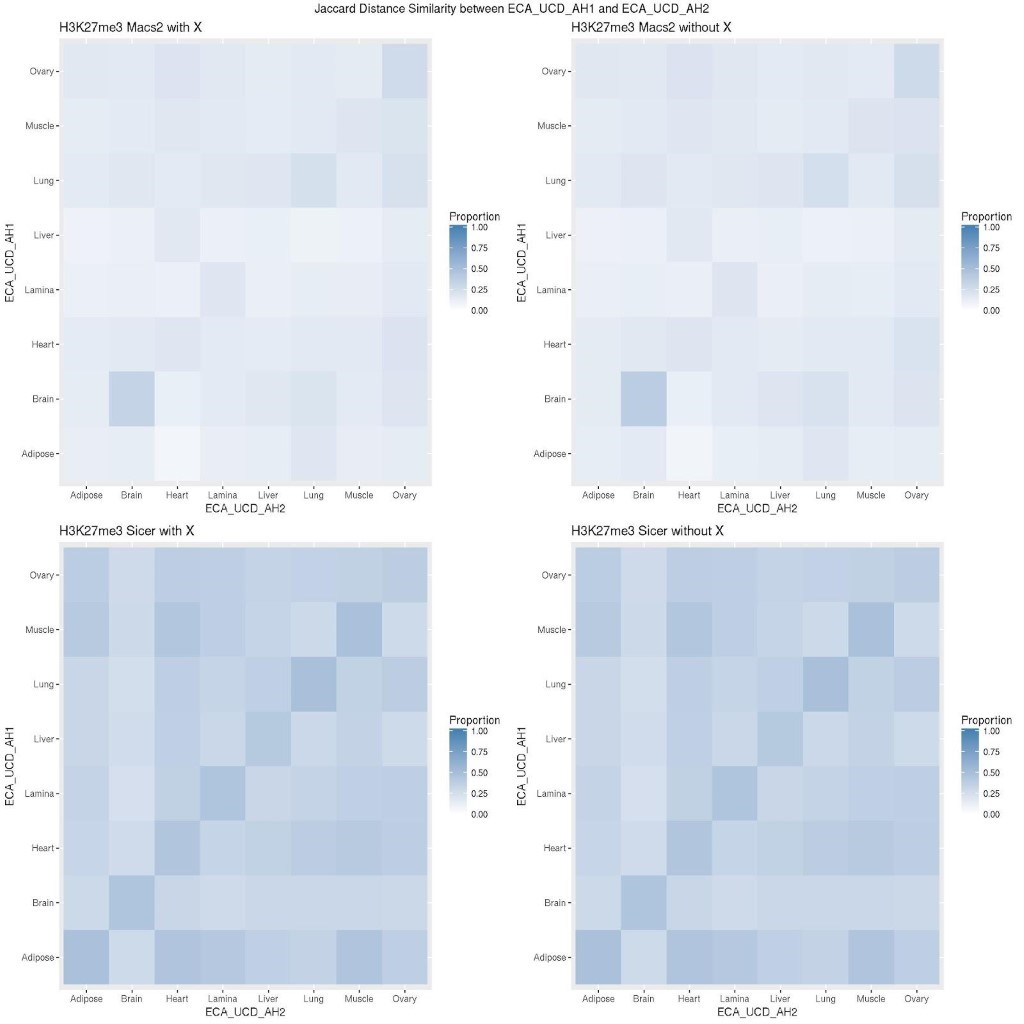


**Figure S1: Jaccard Index to measure similarity of peaks called for H3K27me3 based on either MACS2 or SICER peak-calling algorithms.** Each panel is a heatmap displaying the Jaccard Index, from pairwise comparisons of tissues between ECA_UCD_AH1 and ECA_UCD_AH2. Darker blue indicates that there are more peaks that are shared by the two samples. The top row compares peaks called by MACS2 including the X chromosome (Panel 1) and without the repressed X chromosome (Panel 2). The bottom row compares peaks called by SICER including the X chromosome (Panel 3) and without the X chromosome (Panel 4).

**Supplemental Table Descriptions:**

**Table S1: Concentration and catalog numbers for antibodies used during ChIP.**

**Table S2: Quality metrics and bioinformatic summary of QC testing.** First tab lists the alignment summary organized by histone mark, and second tab lists the alignment summary organized by tissue. Third tab lists the six quality metrics organized by histone mark, and fourth tab lists the six quality metrics organized by tissue.

**Table S3: Comparing peak numbers to ENCODE data.** All peaks were called with MACS2 software. For combined peak calls for H3K27me3 by SICER, see Table 3. For ENCODE data, all samples were from adult females of middle age, except for brain (temporal lobe) which was from an elderly female individual. The same adult female biosample was used where possible, and ENCODE data are peaks called from one biological sample.

**Table S4: Motif and GO term analysis results for adipose-specific active enhancers.** Active enhancers were defined by overlap of H3K4me1 and H3K27ac marks without overlap by H3K27me3 or H3K4me3. Tissue-specificity was defined as a region that was identified in only one of the eight tissues of interest. First tab (labelled ame) lists the AME results from MEME suite. Second tab (labelled Uniprot) contains the Swiss Prot entries for each transcription factor implicated by AME for any organism. Third tab (labelled Summary) contains the summary of the Swiss Prot information from human research (or mouse if human was not available) for each transcription factor implicated by AME. The Biological Process GO terms are listed in separate cells rather than a semicolon separate list as in the second tab.

**Table S5: Motif and GO term analysis results for adipose-specific active promoters.** Active promoters were defined by overlap of H3K4me3 and H3K27ac marks without overlap by H3K27me3. Tissue-specificity was defined as a region that was identified in only one of the eight tissues of interest. First tab (labelled ame) lists the AME results from MEME suite. Second tab (labelled Uniprot) contains the Swiss Prot entries for each transcription factor implicated by AME for any organism. Third tab (labelled Summary) contains the summary of the Swiss Prot information from human research (or mouse if human was not available) for each transcription factor implicated by AME. The Biological Process GO terms are listed in separate cells rather than a semicolon separate list as in the second tab.

**Table S6: Motif and GO term analysis results for brain-specific active enhancers.** Active enhancers were defined by overlap of H3K4me1 and H3K27ac marks without overlap by H3K27me3 or H3K4me3. Tissue-specificity was defined as a region that was identified in only one of the eight tissues of interest. First tab (labelled ame) lists the AME results from MEME suite. Second tab (labelled Uniprot) contains the Swiss Prot entries for each transcription factor implicated by AME for any organism. Third tab (labelled Summary) contains the summary of the Swiss Prot information from human research (or mouse if human was not available) for each transcription factor implicated by AME. The Biological Process GO terms are listed in separate cells rather than a semicolon separate list as in the second tab.

**Table S7: Motif and GO term analysis results for brain-specific active promoters.** Active promoters were defined by overlap of H3K4me3 and H3K27ac marks without overlap by H3K27me3. Tissue-specificity was defined as a region that was identified in only one of the eight tissues of interest. First tab (labelled ame) lists the AME results from MEME suite. Second tab (labelled Uniprot) contains the Swiss Prot entries for each transcription factor implicated by AME for any organism. Third tab (labelled Summary) contains the summary of the Swiss Prot information from human research (or mouse if human was not available) for each transcription factor implicated by AME. The Biological Process GO terms are listed in separate cells rather than a semicolon separate list as in the second tab.

**Table S8: Motif and GO term analysis results for heart-specific active enhancers.** Active enhancers were defined by overlap of H3K4me1 and H3K27ac marks without overlap by H3K27me3 or H3K4me3. Tissue-specificity was defined as a region that was identified in only one of the eight tissues of interest. First tab (labelled ame) lists the AME results from MEME suite. Second tab (labelled Uniprot) contains the Swiss Prot entries for each transcription factor implicated by AME for any organism. Third tab (labelled Summary) contains the summary of the Swiss Prot information from human research (or mouse if human was not available) for each transcription factor implicated by AME. The Biological Process GO terms are listed in separate cells rather than a semicolon separate list as in the second tab.

**Table S9: Motif and GO term analysis results for heart-specific active promoters.** Active promoters were defined by overlap of H3K4me3 and H3K27ac marks without overlap by H3K27me3. Tissue-specificity was defined as a region that was identified in only one of the eight tissues of interest. First tab (labelled ame) lists the AME results from MEME suite. Second tab (labelled Uniprot) contains the Swiss Prot entries for each transcription factor implicated by AME for any organism. Third tab (labelled Summary) contains the summary of the Swiss Prot information from human research (or mouse if human was not available) for each transcription factor implicated by AME. The Biological Process GO terms are listed in separate cells rather than a semicolon separate list as in the second tab.

**Table S10: Motif and GO term analysis results for lamina-specific active enhancers.** Active enhancers were defined by overlap of H3K4me1 and H3K27ac marks without overlap by H3K27me3 or H3K4me3. Tissue-specificity was defined as a region that was identified in only one of the eight tissues of interest. First tab (labelled ame) lists the AME results from MEME suite. Second tab (labelled Uniprot) contains the Swiss Prot entries for each transcription factor implicated by AME for any organism. Third tab (labelled Summary) contains the summary of the Swiss Prot information from human research (or mouse if human was not available) for each transcription factor implicated by AME. The Biological Process GO terms are listed in separate cells rather than a semicolon separate list as in the second tab.

**Table S11: Motif and GO term analysis results for lamina-specific active promoters.** Active promoters were defined by overlap of H3K4me3 and H3K27ac marks without overlap by H3K27me3. Tissue-specificity was defined as a region that was identified in only one of the eight tissues of interest. First tab (labelled ame) lists the AME results from MEME suite. Second tab (labelled Uniprot) contains the Swiss Prot entries for each transcription factor implicated by AME for any organism. Third tab (labelled Summary) contains the summary of the Swiss Prot information from human research (or mouse if human was not available) for each transcription factor implicated by AME. The Biological Process GO terms are listed in separate cells rather than a semicolon separate list as in the second tab.

**Table S12: Motif and GO term analysis results for liver-specific active enhancers.** Active enhancers were defined by overlap of H3K4me1 and H3K27ac marks without overlap by H3K27me3 or H3K4me3. Tissue-specificity was defined as a region that was identified in only one of the eight tissues of interest. First tab (labelled ame) lists the AME results from MEME suite. Second tab (labelled Uniprot) contains the Swiss Prot entries for each transcription factor implicated by AME for any organism. Third tab (labelled Summary) contains the summary of the Swiss Prot information from human research (or mouse if human was not available) for each transcription factor implicated by AME. The Biological Process GO terms are listed in separate cells rather than a semicolon separate list as in the second tab.

**Table S13: Motif and GO term analysis results for liver-specific active promoters.** Active promoters were defined by overlap of H3K4me3 and H3K27ac marks without overlap by H3K27me3. Tissue-specificity was defined as a region that was identified in only one of the eight tissues of interest. First tab (labelled ame) lists the AME results from MEME suite. Second tab (labelled Uniprot) contains the Swiss Prot entries for each transcription factor implicated by AME for any organism. Third tab (labelled Summary) contains the summary of the Swiss Prot information from human research (or mouse if human was not available) for each transcription factor implicated by AME. The Biological Process GO terms are listed in separate cells rather than a semicolon separate list as in the second tab.

**Table S14: Motif and GO term analysis results for lung-specific active enhancers.** Active enhancers were defined by overlap of H3K4me1 and H3K27ac marks without overlap by H3K27me3 or H3K4me3. Tissue-specificity was defined as a region that was identified in only one of the eight tissues of interest. First tab (labelled ame) lists the AME results from MEME suite. Second tab (labelled Uniprot) contains the Swiss Prot entries for each transcription factor implicated by AME for any organism. Third tab (labelled Summary) contains the summary of the Swiss Prot information from human research (or mouse if human was not available) for each transcription factor implicated by AME. The Biological Process GO terms are listed in separate cells rather than a semicolon separate list as in the second tab.

**Table S15: Motif and GO term analysis results for lung-specific active promoters.** Active promoters were defined by overlap of H3K4me3 and H3K27ac marks without overlap by H3K27me3. Tissue-specificity was defined as a region that was identified in only one of the eight tissues of interest. First tab (labelled ame) lists the AME results from MEME suite. Second tab (labelled Uniprot) contains the Swiss Prot entries for each transcription factor implicated by AME for any organism. Third tab (labelled Summary) contains the summary of the Swiss Prot information from human research (or mouse if human was not available) for each transcription factor implicated by AME. The Biological Process GO terms are listed in separate cells rather than a semicolon separate list as in the second tab.

**Table S16: Motif and GO term analysis results for muscle-specific active enhancers.** Active enhancers were defined by overlap of H3K4me1 and H3K27ac marks without overlap by H3K27me3 or H3K4me3. Tissue-specificity was defined as a region that was identified in only one of the eight tissues of interest. First tab (labelled ame) lists the AME results from MEME suite. Second tab (labelled Uniprot) contains the Swiss Prot entries for each transcription factor implicated by AME for any organism. Third tab (labelled Summary) contains the summary of the Swiss Prot information from human research (or mouse if human was not available) for each transcription factor implicated by AME. The Biological Process GO terms are listed in separate cells rather than a semicolon separate list as in the second tab.

**Table S17: Motif and GO term analysis results for muscle-specific active promoters.** Active promoters were defined by overlap of H3K4me3 and H3K27ac marks without overlap by H3K27me3. Tissue-specificity was defined as a region that was identified in only one of the eight tissues of interest. First tab (labelled ame) lists the AME results from MEME suite. Second tab (labelled Uniprot) contains the Swiss Prot entries for each transcription factor implicated by AME for any organism. Third tab (labelled Summary) contains the summary of the Swiss Prot information from human research (or mouse if human was not available) for each transcription factor implicated by AME. The Biological Process GO terms are listed in separate cells rather than a semicolon separate list as in the second tab.

**Table S18: Motif and GO term analysis results for ovary-specific active enhancers.** Active enhancers were defined by overlap of H3K4me1 and H3K27ac marks without overlap by H3K27me3 or H3K4me3. Tissue-specificity was defined as a region that was identified in only one of the eight tissues of interest. First tab (labelled ame) lists the AME results from MEME suite. Second tab (labelled Uniprot) contains the Swiss Prot entries for each transcription factor implicated by AME for any organism. Third tab (labelled Summary) contains the summary of the Swiss Prot information from human research (or mouse if human was not available) for each transcription factor implicated by AME. The Biological Process GO terms are listed in separate cells rather than a semicolon separate list as in the second tab.

**Table S19: Summary of the GO term analysis results for unique active elements from all tissues.** First tab (labelled Promoters) lists all of the transcription factors implicated in the active promoters for all of the tissues. Second tab (labelled Enhancers) lists all of the transcription factors implicated in the active enhancers for all of the tissues. A novel motif was defined as a motif that was not identified by AME based on the JASPAR 2018 Vertebrate database but was identified by MEME or DREME (from the MEME Suite) and was not found in any other database based on manual curation. It is unclear at this time if these novel motifs have a functional role.
